# Correlation between particle size/domain structure and magnetic properties of highly crystalline Fe_3_O_4_ nanoparticles

**DOI:** 10.1038/s41598-017-09897-5

**Published:** 2017-08-30

**Authors:** Qing Li, Christina W. Kartikowati, Shinji Horie, Takashi Ogi, Toru Iwaki, Kikuo Okuyama

**Affiliations:** 1 Department of Environmental Science and Engineering, Fudan University, Shanghai, 200433 China; 2 Department of Chemical Engineering, Graduate School of Engineering, Hiroshima University, 1-4-1 Kagamiyama, Higashi, Hiroshima 739-8527 Japan; 3 Technical Strategy Department, Research and Development Division, Toda Kogyo Corporation, Otake, Hiroshima 739-0652 Japan

## Abstract

Highly crystalline single-domain magnetite Fe_3_O_4_ nanoparticles (NPs) are important, not only for fundamental understanding of magnetic behaviour, but also for their considerable potential applications in biomedicine and industry. Fe_3_O_4_ NPs with sizes of 10–300 nm were systematically investigated to reveal the fundamental relationship between the crystal domain structure and the magnetic properties. The examined Fe_3_O_4_ NPs were prepared under well-controlled crystal growth conditions using a large-scale liquid precipitation method. The crystallite size of cube-like NPs estimated from X-ray diffraction pattern increased linearly as the particle size (estimated by transmission electron microscopy) increased from 10 to 64.7 nm, which indicates that the NPs have a single-domain structure. This was further confirmed by the uniform lattice fringes. The critical size of approximately 76 nm was obtained by correlating particle size with both crystallite size and magnetic coercivity; this was reported for the first time in this study. The coercivity of cube-like Fe_3_O_4_ NPs increased to a maximum of 190 Oe at the critical size, which suggests strong exchange interactions during spin alignment. Compared with cube-like NPs, sphere-like NPs have lower magnetic coercivity and remanence values, which is caused by the different orientations of their polycrystalline structure.

## Introduction

Magnetite (Fe_3_O_4_) nanoparticles (NPs) have attracted considerable interest as a result of their attractive properties, such as their strong magnetic moment, biocompatibility, chemical stability, and magnetoelectric properties^1^. Numerous reports have described their advances in nanotechnology and their development of a wide range of applications, such as medical application^2^, catalysis^3^, Li batteries^4^, printer toners^5^, and wastewater treatment^6^. For the broad application of these NPs, control of the crystal size, shape, and domain structure is important for defining their chemical and physical properties^7^. This was demonstrated in our previous study^8^ of photoluminescent NPs, for which the performance was attributable to the domain size rather than the particle size. The particle size, which originates from the magnetic domain structure, has also been recognised as a key factor in the application of magnetic NPs.

The relationship between particle size and the magnetic properties, such as the coercivity (*Hc*), of Fe_3_O_4_ NPs has been widely reported. The critical size of magnetic NPs, which indicates the transition from a single- to multi-domain structure, was evaluated by the change in *Hc* with respect to the particle size. However, the critical size for Fe_3_O_4_ NPs has not yet been systematically demonstrated because this value depends on the crystal structure, which can have spherical, cubic, or multiple phases. Therefore, investigation of the critical size on the basis of the crystal structure is necessary.

The particle size required to achieve superparamagnetism in Fe_3_O_4_ NPs is widely estimated to be below 20 nm^6, 9–11^, whereas the critical size for forming a multi-domain structure has been theoretically estimated to be 76 nm for cubic^12^ and 128 nm for spherical Fe_3_O_4_ NPs^13^. However, the critical size for cubic Fe_3_O_4_ NPs has been experimentally determined to be higher than 160 nm^14^. Much smaller critical sizes of 30–46 nm^15–18^ have also been reported for cubic Fe_3_O_4_ NPs. Multi-granule Fe_3_O_4_ NPs with sizes of 16–512 nm showed a transitional size of approximately 120 nm^19^. Although the effects of size and shape on the behaviour of magnetic particles have been known for more than half a century^10^, the quantitative effects on Zn_0.4_Fe_2.6_O_4_ NPs remained undiscovered in the size range of 20–140 nm until 2012^20^, when the critical size was found to be approximately 60 nm, and the saturation magnetisation (*Ms*) was found to be lower for spherical particles than for cubic ones.

Owing to the experimental difficulty in controlling particle sizes over a wide size range^21^, a systematic investigation of the magnetic domain structures of the most commonly used Fe_3_O_4_ NPs is still lacking, although such an investigation is needed to meet the currently increasing requirements for various applications. The current study therefore investigates the size dependence of the magnetic properties of Fe_3_O_4_ NPs with sizes of 10‒300 nm. The study includes cube- and sphere-like NPs that were produced under well-controlled crystal growth conditions on a large scale. The critical size of highly crystalline cube-like Fe_3_O_4_ NPs was examined through the correlation between the particle size measured by transmission electron microscopy (TEM) and the crystallite size estimated by X-ray diffraction (XRD). The value was further confirmed by observing the lattice fringes and examining the dependence of *Hc* on the particle size. The high *Hc* value obtained in this study is discussed in detail in terms of the spin interactions in a single-domain structure.

## Methods

Two types of highly crystalline Fe_3_O_4_ NPs, i.e., cube-like and sphere-like ones, were synthesised on a large scale under precise control of the Fe^2+^ concentration, pH, temperature, and aeration rate. The cube-like Fe_3_O_4_ NPs were prepared by a two-stage oxidation reaction, which is described in patent No. US 5843610A (Toda Kogyo Co., Ltd., Japan)^22^. In contrast, the sphere-like Fe_3_O_4_ NPs were prepared by a one-stage oxidation reaction, which is described in patent No. US 4992191A (Toda Kogyo Co., Ltd., Japan)^23^. All data generated or analyzed during this study are included in this article and its Supplementary Information files.

The morphologies of the prepared NPs were analysed using field-emission scanning electron microscopy (FE SEM; Hitachi S-5000, Tokyo, Japan) and TEM (JEM-2010, 200 kV, JEOL Ltd., Tokyo, Japan). The crystallite size and chemical composition of the prepared NP samples were examined by XRD (RINT2000, Rigaku Denki Co. Ltd., Tokyo, Japan), using Cu Kα radiation with a scanning range of 2*θ* 10–80°. Their magnetic performance was assessed using a superconducting quantum interference device (SQUID, Quantum Design, Tokyo, Japan), operated at 300 K. The prepared cube-like NPs with particle sizes (*d*_p_) of 9.6, 19.6, 24.4, 31.9, 45.3, 64.7, 130, 243, and 287 nm were named as C1, C2, C3, C4, C5, C6, C7, C8, and C9, respectively. The sphere-like NPs with *d*_p_ of 93.3 and 121 nm were named as S1 and S2, respectively.

## Results and Discussion

**Figure 1:**
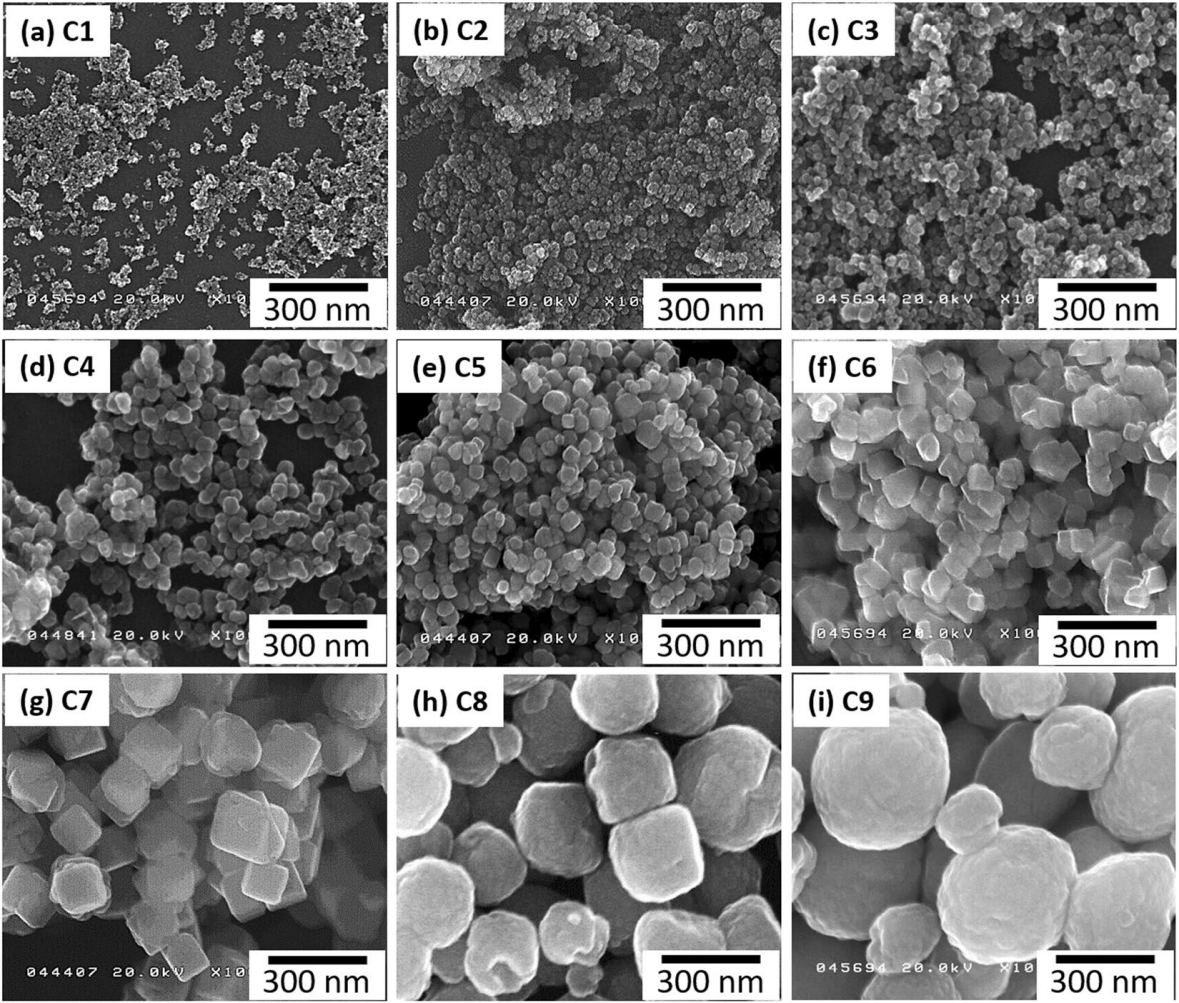
Scanning electron microscopy images of cube-like Fe_3_O_4_ nanoparticles with various particle sizes. (**a**) 9.6, (**b**) 19.6, (**c**) 24.4, (**d**) 31.9, (**e**) 45.3, (**f**) 64.7, (**g**) 130, (**h**) 243, and (**i**) 287 nm, which are named as C1‒C9, respectively.

SEM images of the cube-like Fe_3_O_4_ NPs are shown in Fig. 1. The *d*_p_ of these particles are 9.6, 19.6, 24.4, 31.9, 45.3, 64.7, 130, 243, and 287 nm for C1‒C9, respectively, and their size distributions are given in Supplementary Fig. S1. The high-resolution TEM (HRTEM) images of the cube-like Fe_3_O_4_ NPs in Supplementary Fig. S2 show the lattice fringes of the NPs, and indicate that the NPs have a single crystalline structure up to a *d*_p_ of 64.7 nm. In contrast, the C7–C9 NPs, with diameters larger than 100 nm, show a polycrystalline structure. SEM images and size distributions of the sphere-like Fe_3_O_4_ NPs are shown in Supplementary Fig. S3(a–d). The values of *d*_p_ were 93.3 and 121 nm for S1 and S2, respectively. All of the Fe_3_O_4_ NPs exhibit quasi-narrow size distributions compared with previously reported particles^7, 14^.

**Figure 2:**
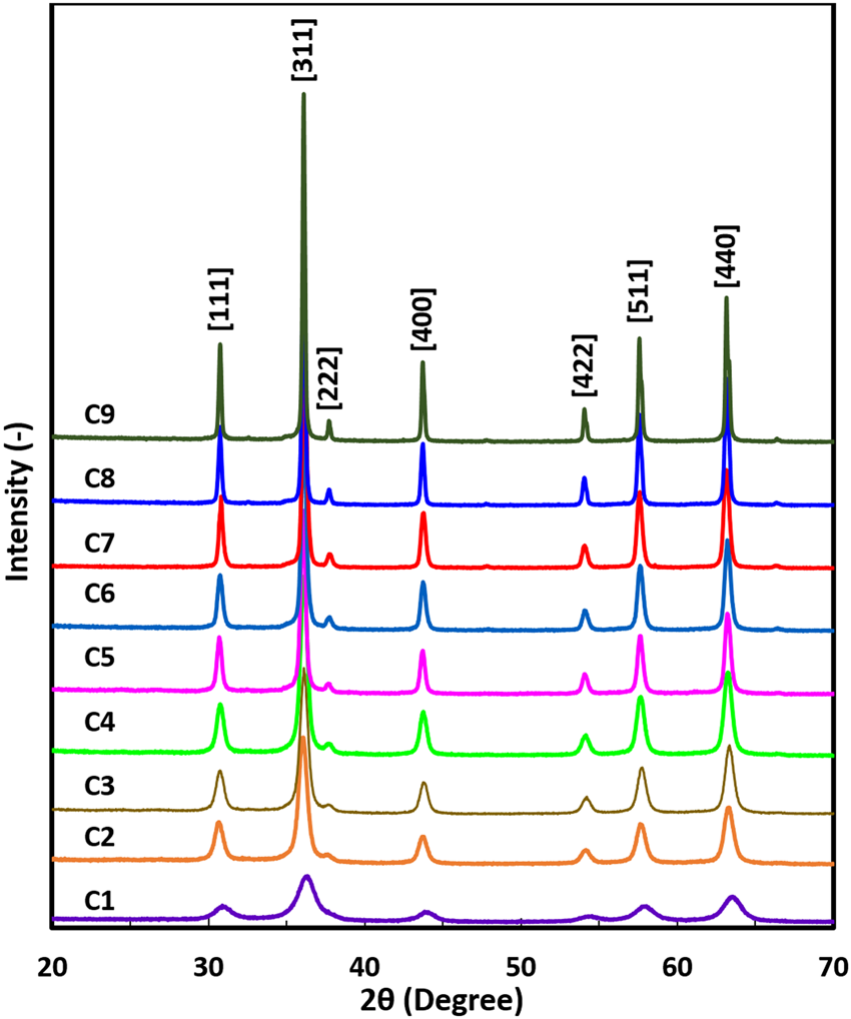
X-ray diffraction patterns of cube-like Fe_3_O_4_ nanoparticles with various particle sizes.

The XRD patterns of cube-like Fe_3_O_4_ NPs are shown in Fig. 2. The diffraction peaks of 2*θ* correspond to the [111], [311], [222], [400], [422], [511], and [440] planes, which indicate the pure cubic phase of the Fe_3_O_4_ NPs. The sharp peaks indicate the highly crystalline nature of the Fe_3_O_4_ NPs. The XRD patterns of the sphere-like NPs, shown in Supplementary Fig. S3(e), also demonstrate the high crystallinity of these particles. Impurity peaks and transition phases were not observed, which indicates that the particles prepared by the liquid precipitation method were pure, both chemically and in their crystalline phase. The crystallite sizes (*d*_c_) of all the Fe_3_O_4_ NPs were estimated by the Scherrer formula using the highest intensity XRD peak^24^, namely [311], and compared with the *d*_p_ obtained from the TEM analysis, as listed in Supplementary Table S1.

**Figure 3:**
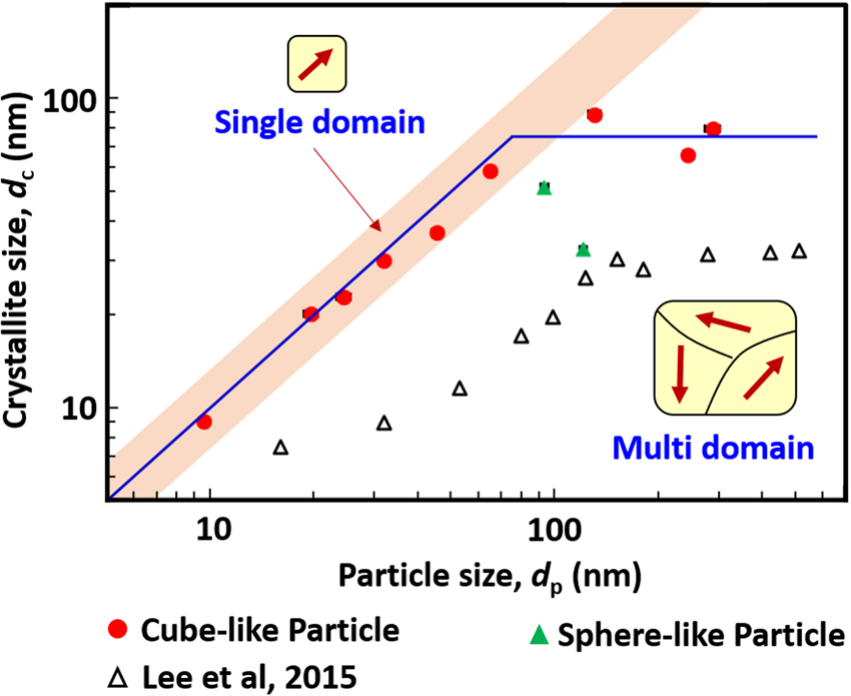
Relationship between average particle size and crystallite size. The solid line shows the trend in the relationship for cube-like Fe_3_O_4_ nanoparticles. The black open triangles were replotted from Table 1 in ref. 19 for multi-granule Fe_3_O_4_ nanoparticles.

The relationship between *d*_p_ and *d*_c_ is shown in Fig. 3. The straight line indicates a single crystalline particle, whereas the particles corresponding to points that fall below this line are polycrystalline. Notably, the *d*_c_ and corresponding *d*_p_ have almost the same value for particles with a diameter of 10 to around 80 nm, which indicates a single-domain structure. The particles with diameters of larger than 80 nm have a constant *d*_c_, which indicates a multi-domain structure. The critical size of these cube-like NPs was calculated as 78 ± 9 nm from the relationship between *d*_p_ and *d*_c_. The critical size is usually obtained from the change in magnetic properties, such as the relationship of *Hc* and *d*_p_, and this is the first time it has been obtained from the relationship between *d*_p_ and *d*_c_ for Fe_3_O_4_ NPs. The same tendency was observed by Lee *et al*.^19^ in 2015 for multi-granule Fe_3_O_4_ NPs, which have a smaller *d*_c_ than our NPs. The HRTEM images shown in Supplementary Fig. S2 confirm that NPs with a diameter of up to 64.7 nm have a single crystalline structure, which is shown by the single direction of the lattice fringes. This result is consistent with those obtained for particles produced by a colloidal chemical synthetic route^7, 14^.

The sphere-like NPs show different crystal properties. For these NPs, the *d*_c_ is much smaller than the *d*_p_, and also much smaller than those of the cube-like NPs with a similar *d*_p_, as shown in Supplementary Table S1 and Fig. 3. Supplementary Fig. S4 shows a detailed comparison of the cube-like C7 and sphere-like S2 NPs, which have a similar average *d*_p_. The dark-field TEM image and electron diffraction pattern show that the C7 NP is polycrystalline with a single orientation. However, a polycrystalline structure containing different orientations was observed in the S2 NPs. This was further confirmed from the HRTEM images by the existence of different directions of the lattice fringe in a single S2 NP (Supplementary Fig. S4(g–i)). Sphere-like NPs commonly consist of agglomerates of variously sized cubic NPs^5, 14, 25^. The different morphological structure arises from the different preparation processes^11^, as described in the patent^22, 23^.

**Figure 4:**
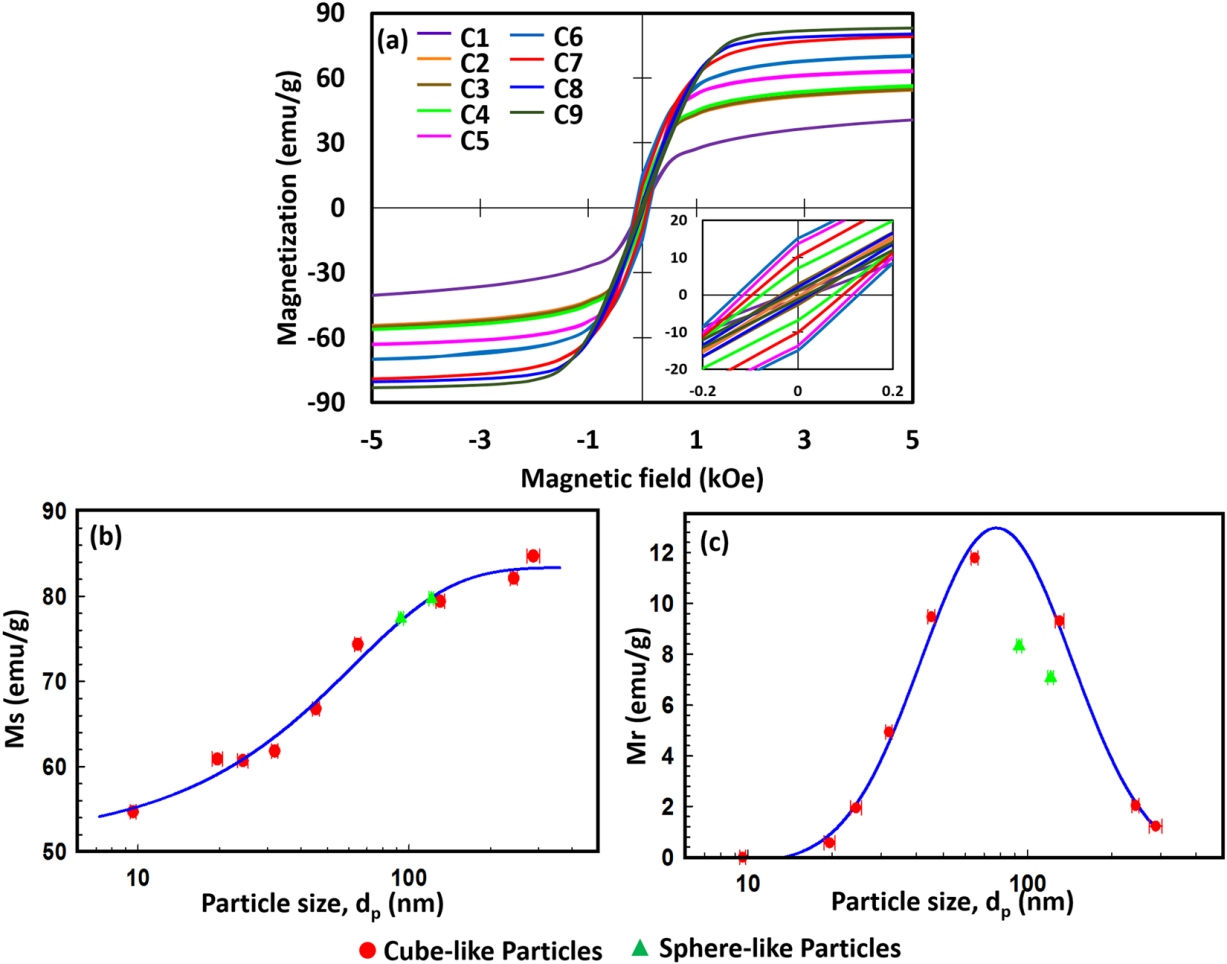
(**a**) Hysteresis loops of cube-like Fe_3_O_4_ nanoparticles, and particle size dependence of (**b**) saturation magnetization and (**c**) remanent magnetization for all Fe_3_O_4_ nanoparticles.

**Figure 5:**
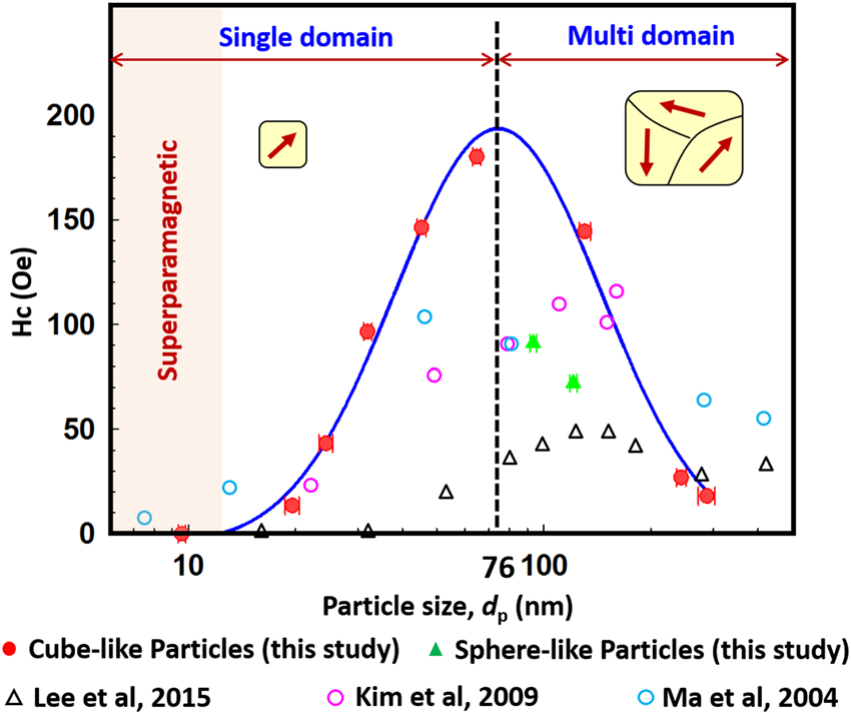
Magnetic performance of Fe_3_O_4_ nanoparticles measured at 300 K: particle size dependence of the coercivity (*Hc*). The black open triangles, violet open circles, and blue open circles were replotted from refs 14, 15 and 19, respectively.

The hysteresis loops for these particles, shown in Fig. 4(a), show the ferrimagnetic nature of the Fe_3_O_4_ NPs. The cube-like Fe_3_O_4_ NPs possess high *Ms* values, which are affected by the *d*_p_ as shown in Fig. 4(b). The *Ms* value, obtained by applying the law of approach to saturation^26^, increases with increasing *d*_p_ for all samples, including the sphere-like NPs. This trend is consistent with those found in other reports on Fe_3_O_4_ NPs with diameters lower than 100 nm^5, 16, 27, 28^. The *Ms* value increased from 54.7 emu/g (9.6-nm NPs) to 84.7 emu/g (287-nm NPs), which is close to the theoretically estimated *Ms* for bulk Fe_3_O_4_ (92 emu/g). The *Hc* and remanent magnetisation (*Mr*), which are also affected by *d*_p_, are shown in Figs 4(c) and 5. These values increase from around 0 for the 9.6-nm NPs, which are known to be superparamagnetic^26, 29, 30^, to a maximum value of around 190 Oe (*Hc*) and 13 emu/g (*Mr*) at a *d*_p_ of around 80 nm, and then decrease continuously with further increases in *d*_p_. The trends in these two parameters are consistent with previous theoretical estimations^10, 19, 31^, and they have similar characteristics to those obtained for Zn_0.4_Fe_2.6_O_4_ NPs^20^. The initial increase in *Hc* with domain size corresponds to the sixth power of the domain size. The high *Hc* value may be caused by the strong spin interactions in highly crystalline Fe_3_O_4_ NPs during spin alignment, which has previously been observed in soft magnetic NPs^32^.

By fitting the measured *Mr* and *Hc* values using a log-normal distribution function, shown by the dark blue solid line, the critical sizes for the maximum *Mr* and *Hc* values were determined to be 77 ± 2 nm and 75 ± 3 nm, respectively. On average, therefore, the critical value for the transition is about 76 ± 4 nm. This value is consistent with the critical size of 76 nm estimated theoretically for the transition from single- to multi-domain behavior^12^. Furthermore, the critical size of about 76 nm is almost the same as the transition size obtained from the relationship of *d*_c_ and *d*_p_, as shown in Fig. 3. Single-domain cube-like Fe_3_O_4_ NPs, such as those with a size of 64.7 nm, can be applied effectively as starting materials for many real products, especially for new rare-earth-free magnets with high magnetic moments, by transformation into α″-Fe_16_N_2_ NPs and subsequent dispersion and assembly under a magnetic field^33–38^.

The crystalline properties of the NPs affect their magnetic properties, especially *Hc*. The different crystalline properties of the cube- and sphere-like NPs result in their different magnetic performances, as shown in Figs 4(c) and 5. The measured *Mr* and *Hc* values for the sphere-like NPs (S1 and S2) are both lower than the corresponding fitted values for the cube-like NPs. This may be caused by their composite small crystallite size. A comparison of the hysteresis loops of the two NPs with similar *d*_p_ (C7 and S2) in Supplementary Fig. S5 shows the difference between the two samples, although their *Ms* values are similar (79.4 and 79.7 emu/g as listed in Supplementary Table S1). The multiple orientations of the polycrystalline in the sphere-like NPs, which lead to the multiple orientations of their easy axes, is considered to be the reason for their lower *Mr* and *Hc* values compared with those of the cube-like NPs. This is consistent with a previous study on the particle-size and shape dependence of Fe_3_O_4_ NPs^5^. However, further theoretical explanation and experimental investigation are still required.

## Conclusions

The magnetic properties, including the *Ms*, *Mr*, and *Hc*, of Fe_3_O_4_ NPs are highly influenced by the particle size and domain structure. The *Ms* increases with increasing particle size, regardless of the crystal structure and particle shape. After exceeding the superparamagnetic limit, the *Hc* and *Mr* values increase with increasing particle size up to a maximum value of about 190 Oe and 13 emu/g, respectively, at the critical size of 76 nm. Above this critical size, the *Hc* and *Mr* values decrease with further increases in the particle size, and the cube-like Fe_3_O_4_ NPs change from a single- to multi-domain structure. The multiple orientations of the crystallites within the multi-domain-structured NPs lead to the decrease in the *Hc* value. These findings suggest that considerable attention should be given to the particle size and crystalline properties of Fe_3_O_4_ NPs, which have potential biomedical and industrial applications. These applications require that magnetic particles are sized appropriately to achieve a good balance between effective surface area and satisfactory magnetic performance.

## Electronic supplementary material

Supplementary Information
