# Supplementary material for: Correlation between particle size/domain structure and magnetic properties of highly crystalline Fe3O4 nanoparticles
Source: Sci Rep. 2017 Aug 30;7:9894. doi: 10.1038/s41598-017-09897-5 (PMC5577113; doi:10.1038/s41598-017-09897-5)
Supplement: Supplementary file 1 — Supplementary Information [file 41598_2017_9897_MOESM1_ESM.doc]

Supplementary Information

**Correlation between particle size/domain structure and magnetic properties of highly crystalline Fe_3_O_4_ nanoparticles**

Qing Li^1^, Christina W. Kartikowati^2^, Shinji Horie^3^, Takashi Ogi^2*^, Toru Iwaki^2^, and Kikuo Okuyam^2^

^1^ Department of Environmental Science and Engineering, Fudan University, Shanghai

200433, China

^2^ Department of Chemical Engineering, Graduate School of Engineering, Hiroshima University, 1-4-1 Kagamiyama, Higashi-Hiroshima 739-8527, Japan

^3^ Technical Strategy Department, Research and Development Division, Toda Kogyo Corporation, Otake, Hiroshima 739-0652, Japan

^*^E-mail: ogit@hiroshima-u.ac.jp


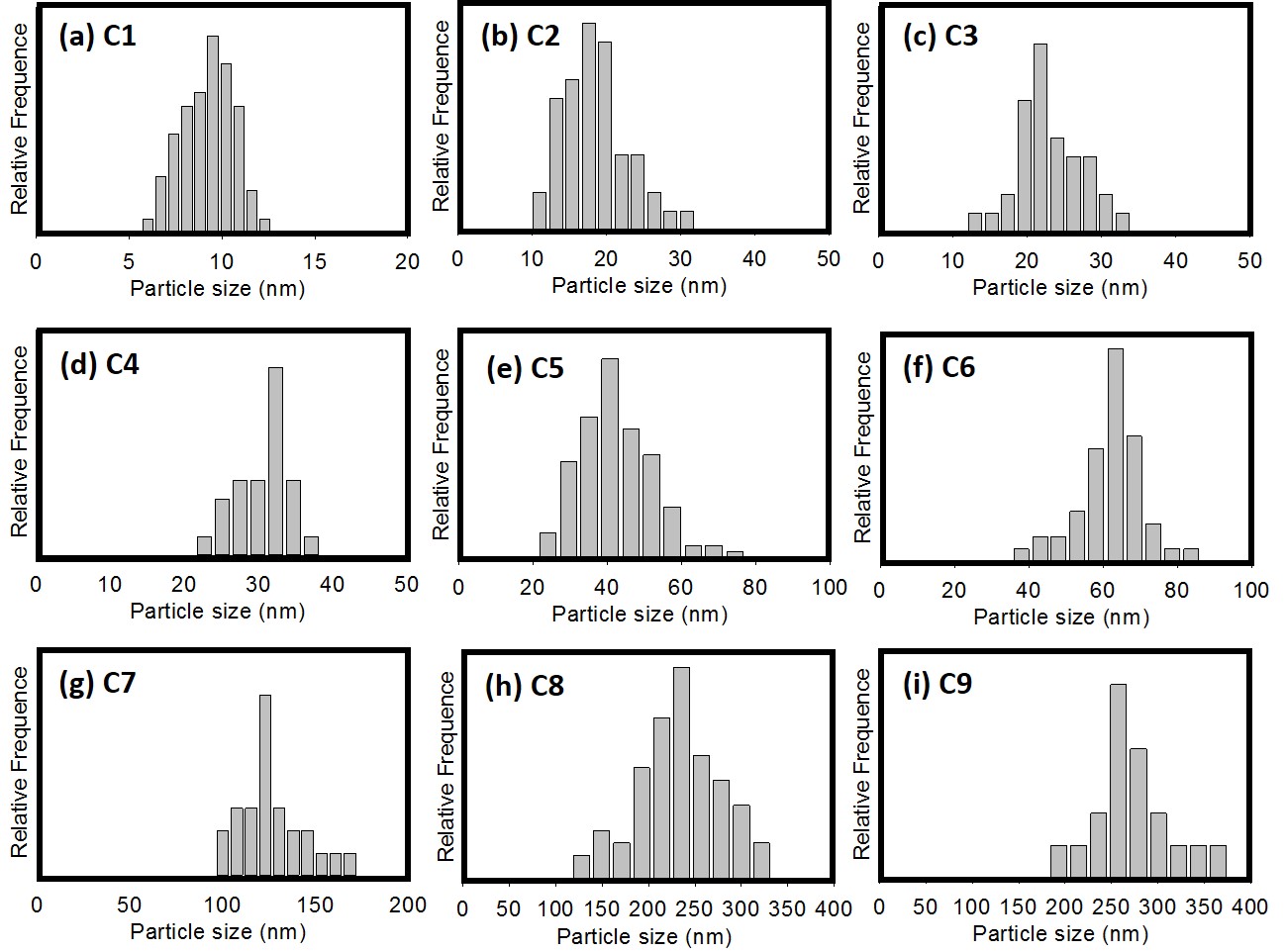


**Supplementary Figure S1.** Size distributions of cube-like Fe_3_O_4_ nanoparticles with various particle sizes. (a) 9.6, (b) 19.6, (c) 24.4, (d) 31.9, (e) 45.3, (f) 64.7, (g) 130, (h) 243, and (i) 287 nm, which are named C1‒C9, respectively.


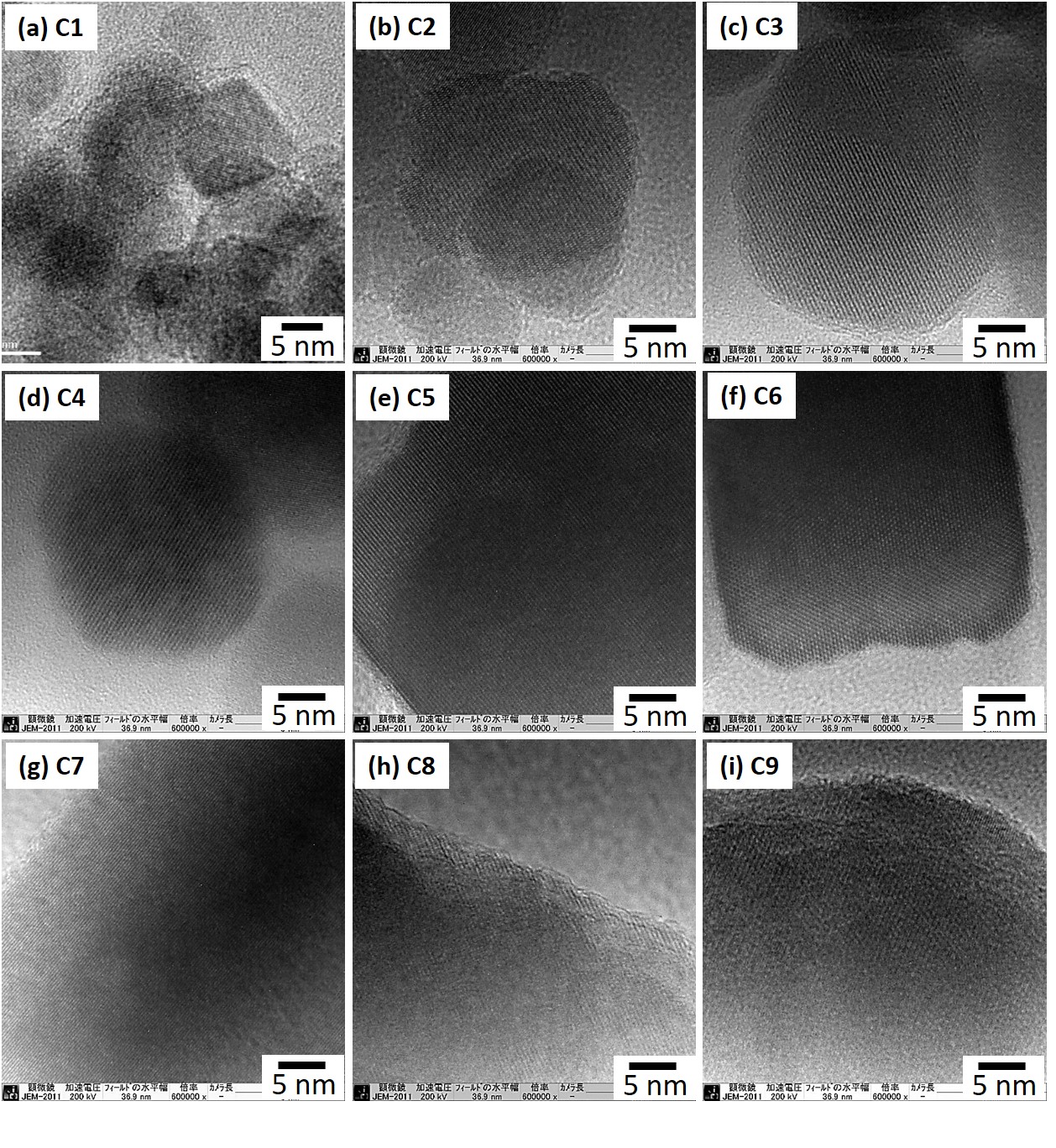


**Supplementary Figure S2.** High resolution transmission electron microscopy images of cube-like Fe_3_O_4_ nanoparticles with various sizes. (a) 9.6, (b) 19.6, (c) 24.4, (d) 31.9, (e) 45.3, (f) 64.7, (g) 130, (h) 243, and (i) 287 nm, which are named C1‒C9, respectively.


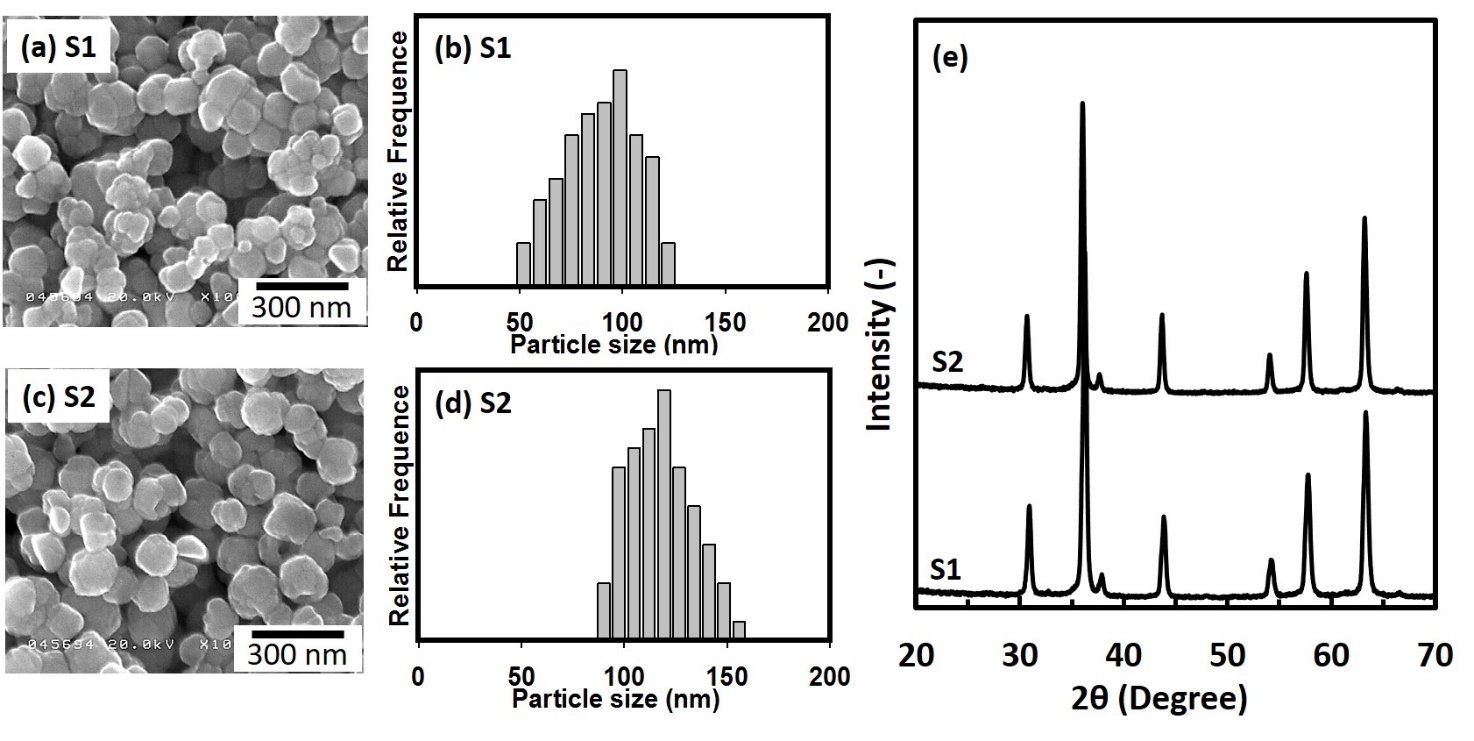


**Supplementary Figure S3.** Particle properties of sphere-like Fe_3_O_4_ nanoparticles S1 (93.3 nm) and S2 (121 nm): (a) and (c) scanning electron microscopy images, (b) and (d) particle size distributions, and (e) XRD patterns.


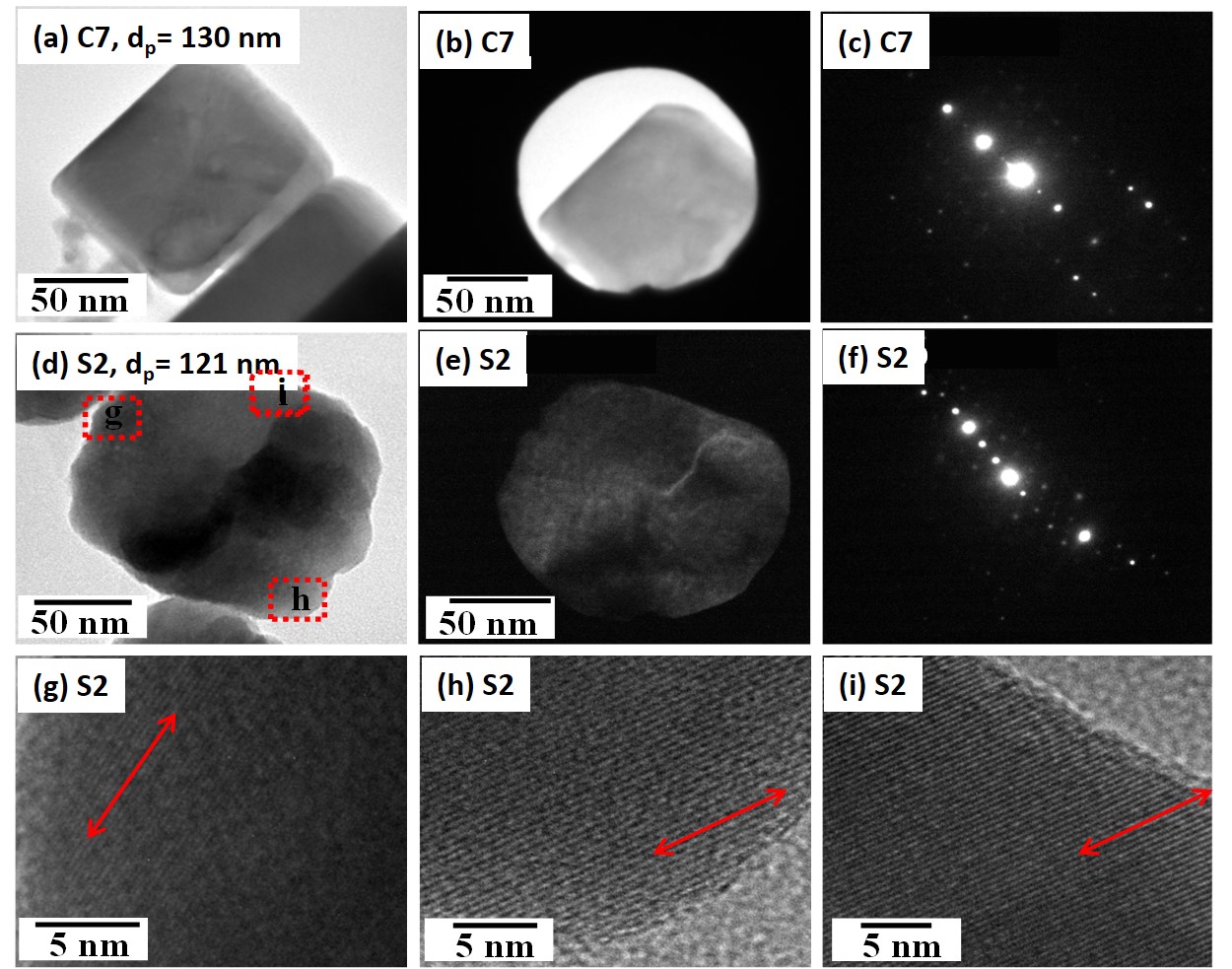


**Supplementary Figure S4.** Comparison of the crystal structures of cube-like C7 and sphere-like S2 nanoparticles: (a) and (d) transmission electron microscopy (TEM) images, (b) and (e) dark-field TEM images, and (c) and (f) single-particle electron diffraction patterns. (g), (h), and (i) are high resolution TEM images of the labelled regions in (d).


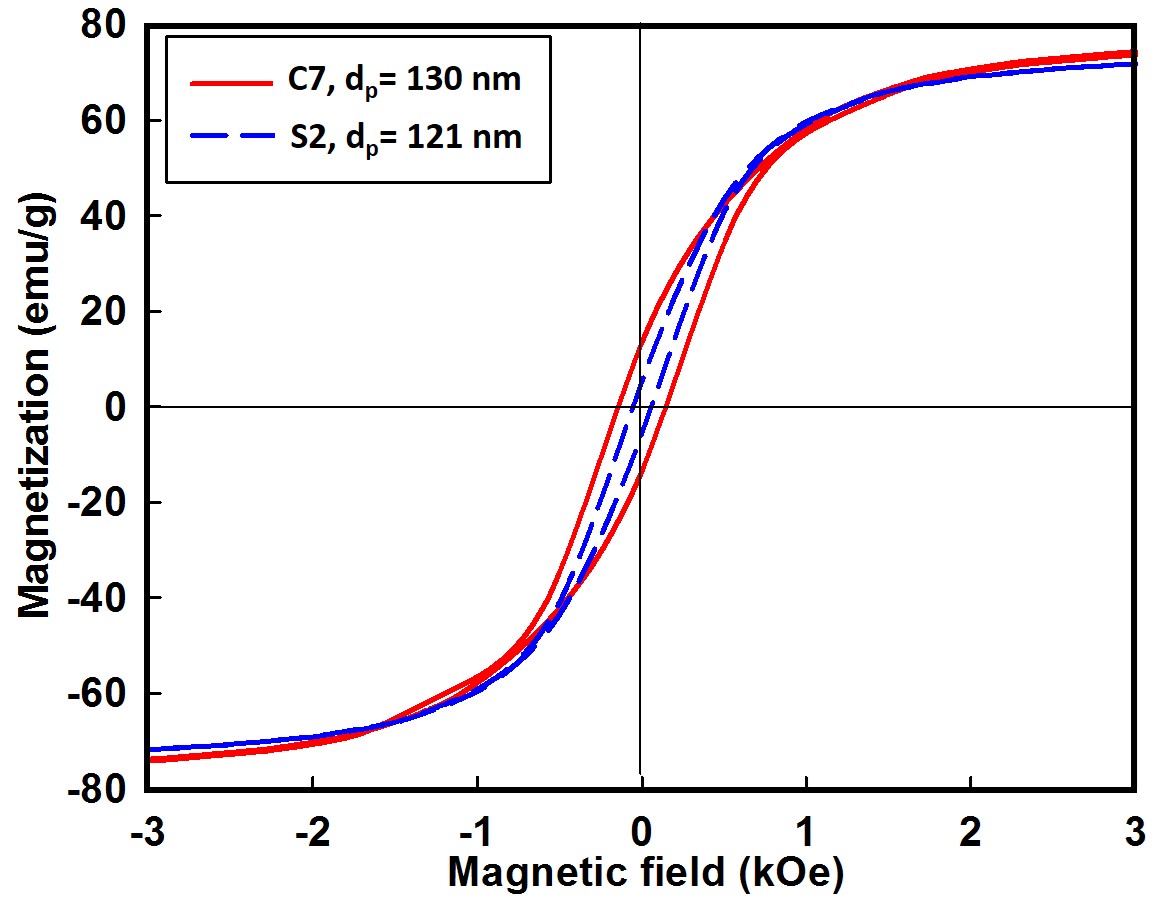


**Supplementary Figure S5.** Comparison of hysteresis loops for cube-like C7 and sphere-like S2 nanoparticles.

**Supplementary Table S1.** Average particle sizes (*d*_p_) estimated from transmission electron microscopy, crystallite sizes (*d*_c_) estimated from X-ray diffraction analysis, and saturation magnetisation (*Ms*) and coercivity (*Hc*) values of the examined Fe_3_O_4_ nanoparticles.

| **Sample** | **Particle size**  **d_p_ (nm)** | **Crystal size**  **d_c_(nm)** | **Saturation magnetization**  **Ms (emu/gr)** | **Coercivity**  **Hc (Oe)** |
| --- | --- | --- | --- | --- |
| C1 | 9.6 | 9.1 | 54.7 | 0 |
| C2 | 19.6 | 20.2 | 60.9 | 13.5 |
| C3 | 24.4 | 22.9 | 60.7 | 43.3 |
| C4 | 31.9 | 29.9 | 61.8 | 96.4 |
| C5 | 45.3 | 36.9 | 66.8 | 146 |
| C6 | 64.7 | 58.2 | 74.4 | 180 |
| C7 | 130 | 88.7 | 79.4 | 144 |
| C8 | 243 | 65.8 | 82.1 | 26.8 |
| C9 | 287 | 79.6 | 84.7 | 18.0 |
| S1 | 93.3 | 51.7 | 77.4 | 90.9 |
| S2 | 121 | 32.4 | 79.7 | 72.0 |
